# Analysis of Gender on Editorial Boards of Sport Sciences Journals

**DOI:** 10.1155/2022/8110135

**Published:** 2022-06-08

**Authors:** Raquel Pastor-Cisneros, Inmaculada Torres Castro, Jose Carmelo Adsuar-Sala, Lucía Bautista-Bárcena

**Affiliations:** ^1^Promoting a Healthy Society Research Group (PHeSO), Faculty of Sport Sciences, University of Extremadura, 10003 Cáceres, Spain; ^2^Department of Mathematics, Faculty of Sport Sciences, University of Extremadura, 10003 Cáceres, Spain; ^3^Department of Mathematics, School of Technology, University of Extremadura, 10003 Cáceres, Spain

## Abstract

This study analyses gender distribution on editorial boards of journals in the *Sport Sciences* category included in Journal Citation Reports (JCR). As far as we know, this study provides the first measure of gender distribution on editorial boards in Sport Sciences. A database consisting of 4,596 editors of journals in the *Sport Sciences* category was collected. The gender of these editors is inferred based on their first name using the package *Gender data* included in the statistical software R. This article found that 23% of the 4,596 editor ships are held by women. This proportion decreases to 10% if we focus on the most important editor of the journal, the so-called Editor in Chief (EiC). However, having a woman EiC is positively correlated with the presence of women on editorial boards. No significant correlation between the impact factor of the journal and the proportion of women editors on its editorial board was found. In addition, most women holding the position of EiC of journals in the first and second quartiles share this position with men.

## 1. Introduction

The under-representation of women in scientific careers is well-known. As a matter of fact, only one in three scientists is a woman in developed countries [1]. Some authors show how research papers produced by women are less read, shared, and cited [2, 3] and they are less likely to receive media coverage [4, 5] than papers produced by men.

In fact, several studies show that it is more difficult for women to achieve scientific eminence [6, 7] than their male colleagues. The inequality between men and women is evident, and this becomes clearer when we progress in the scientific career, where the proportion of women editors in higher positions on the scientific scale is very low.

It is important to notice that women are at a disadvantage with respect to academic publishing, including collaborations, reviews, citations, and media coverage. Men are more likely to collaborate with men, and women are less likely to receive funding when they collaborate with men [8, 9]. Moreover, again in terms of likelihood, the number of women being the first or last author in articles published in more prestigious journals is small [2, 10]. Women are also less likely to be invited to submit papers to journals and act as reviewers [11, 12] and, as a result, men are less likely to respond to requests from women editors to review papers [13, 14]. In addition, women are subject to higher standards of peer review, and therefore, their papers take more than half a year to be published [2]. Given that women are under-represented at so many levels in the scientific environment, one can expect that this under-representation is also found in the composition of the editorial boards of scientific journals. The impact of the editorial boards of scientific journals on the development of science is evident. The editorial board is responsible for organizing their view process of the articles that a journal receives and making decisions on which articles will be published in the journal. The editors contribute to ensuring the quality of the journal, and they have a substantial influence in determining the future direction of the discipline. In the academic field, occupying a position on an editorial board is evidence of a high reputation. Editorial positions are considered prestigious and influential.

The objective of this article was to provide a snapshot of the gender composition of the editorial boards of journals included in the category Sport Sciences (SCIE). The novelty of the subject matter of this article should be emphasized, since this composition had never been analyzed in this area of knowledge.

The fact that women are under-represented in any field of science can lead to various problems: gender gap, pay inequality, lack of women role models for the new generations, etc. Due to the importance of the editorial board position in scientific journals for professional advancement, it is believed that the gender imbalance should be mitigated in order to favor the visualization of women. In order to achieve this objective, it is proposed to

1. Promote policies that favor the inclusion of adverse editorial board in journals by encouraging different publishers to implement it.
2. Reserve resources and incentive research places for women in this field, ensuring their inclusion in research.

For this to happen, it is important to first describe the current position of women in each field of science. The findings are that, in the field of Sport Sciences journals, women represent 23.13% of the editors.

The following distinction is made regarding the concepts of gender and sex. The latter is determined by nature; a person is born with male or female sex. On the other hand, gender, male or female, is learned, and can be educated, changed, and manipulated. Gender is defined as the process by which biologically different individuals become women and men, through the acquisition of attributes that each society defines as belonging to femininity and masculinity. In this sense, gender is the psycho-social construction of the feminine and the masculine.

## 2. Background

In 2016, a study showed that the presence of women in the academic field of mathematics decreased progressively as studies increased in difficulty and specialization, reaching very little female representation in mathematical sciences journal editorships [15]. The study concludes that the degree of under-representation on mathematical sciences journal editorial boards is even more severe than in the field at large. We wanted to extrapolate the study to the case of women in Sport Sciences. There are many similarities with the number of women in mathematics, as sport has been a field traditionally for men for a long time.

This article deals with the representation of gender on the editorial boards of 83 journals of the category *Sport Sciences* (SCIE) included in JCR. These journals belong to 39 different publishers, and the data were gathered from December 2020 to September 2021. It is true that online editorial board information may change at any time, but this study provides a snapshot of the gender composition of the editorial boards at that period of time. As far as we know, in this area of knowledge, this gender composition has never been analyzed before. There are different reasons why it is important to analyze the gender composition of the different editorial boards.

1. Editorial boards will be affected by the under-representation of women who could contribute in a very enriching way by bringing their perspective and experience to the sports area.
2. Membership of the editorial board provides opportunities related to intellectual growth, professional development, and contribution to the decision-making process. Anyone excluded from the editorial board is exempt from access to such benefits or responsibilities.
3. The presence of women on journal editorial boards can also encourage women's advancement in Sport Sciences.
4. Women in editorial positions serve as role models for students.

Previous studies have been carried out on gender representation on editorial boards of journals in areas of knowledge such as mathematics, medicine, or political sciences. *Amazon Mechanical Turk* (Mturk, https://www.mturk.com/) was used in a study where the gender of the mathematics journal editorial board was analyzed [15], due to the enormous number of journals in this field (specifically 314 journals in JCR). This platform is a technological infrastructure that offers simple jobs that a machine cannot do. Mturk makes it easier for individuals and businesses to outsource their processes and jobs to a distributed work force who can perform these tasks virtually: from simple data validation and research to more subjective tasks like survey participation or content moderation.

In this study of 13,067 editors, 90.3% were men versus 8.9% women. For the remaining 0.8%, it was not possible to infer the gender of the editor. What is more, when analyzing 435 journals, 51 of them had no women on their editorial board.

The paper of Morton and Sonnad [16] analyzes the gender composition of editorial boards of medical journals. From a sample of 3,473 editors of 39 editorial boards, 83% of the members of these committees were men. In the field of medical science, Ref. [17] focuses on showing the participation of women on the editorial boards of the 60 top-ranked international journals in this field. The results show that only 15.9% of the positions of EiC are held by a woman. The specialties with the lower rates of representation of women on editorial boards are those that require the greatest responsibility: critical care, anesthesiology, or radiology. In none of the journals in these fields is the position of EiC held by a woman.

In Ref. [18], the composition of editorial boards of 119 psychiatric journals is analyzed, and it was found that women only represent 30.4% of the editorial boards. This article concludes that those journals where women occupy a position of greater power correlate with a reduction in the total percentage of women on the editorial board. In Political Science, it is found that 18% of the positions of EiC are held by women and 23% of the positions of associate editors are held by women [19].

A different approach to the gender perspective in the area of Ecology is shown in the study by Fox *et al.* [13]. It examines a set of data from the peer review process for all papers submitted to the journal *Functional Ecology* from January 2004 to June 2014 to determine how the editor's gender influences the gender balance for reviewer recruitment and how their viewer's gender influences responses to review invitations and the score given to reviewed manuscripts. The proportion of women editors selected to be reviewers decreased if the editor was a man, and the longer the editor's tenure and prestige in the job, the lower the proportion of women editors asked to be reviewers. In addition, women invited to review responded similarly regardless of the gender of the editor, but men invited to review were less likely to accept if the editor was a woman. Mauleón *et al.* [1] focus their study on analyzing the gender of the members of the editorial boards of 131 high-quality Spanish scientific journals, from 1998 to 2009. This study concludes that the presence of women on the editorial boards of the 131 journals varies by area, but in all cases, it is less than 30%. Only 8% of the chief editors were women in 1998, compared to 21% in 2009. It is interesting to compare how the representation of women on editorial boards is dependent on the region. In Mazov *et al.* [20], the authors focus on the absence of women in leading editorial board positions in national journals on Earth sciences, while women occupy leading positions in international journals. Also, in Metz and Harzing [21], Table 1shows a gender difference between Europe and US journal editorial boards.

Considering the importance of scientific journals in the research system, since they are the main communication channel, it is obvious that there is interest in having indicators based on the gender gap of the scientific journals. The group of people included in the editorial board can be analyzed as a marker of gender balance in science.

This article is structured as follows. In the Methodology section, the data collection and the inference process of the editor's gender are explained. The Statistical Analysis and Results section refers to the statistical results of the study, and certain comparisons between journals and publishers related to the proportion of women editors are made. Finally, conclusions and future research lines are shown in the Conclusions, Discussion, and Limitations and Future Research Lines sections.

## 3. Methodology

### 3.1. Data Sources and Study Sample

We merged data from the 83 journals in the category *Sport Sciences* (SCIE) from the Journal Citation Reports (JCR). The information on the members of the editorial board was collected from December 2020 to September 2021 based on the information given in the journals on the website. The year of the JCR data considered is 2018. Several hypothesis tests were carried out to determine the proportion of women in each editorial position in the journal. An extensive comparison was made among the different editorial figures with the data obtained.

The framework of study focuses on tabulating the members of the editorial board of the journals included in this category. In our database, each person appears in the editorial position they hold, whether it is on one or several journals; that is, the data are kept for all those journals in which they hold editorial positions. Regarding editors within the same journal who were in several positions, the lowest position was eliminated to avoid duplication in the database. For example, if “Jane” appears as Editor in Chief, which is the highest position, and in “Editorial Board,” which is generic, “Jane” was kept only in the position of Editor in Chief.

The information collected was the following:

1. Abbreviated name of the journal.
2. First name of the editor.
3. Surname of the editor.
4. Position held on the editorial board.
5. Quartile of the journal.
6. Editorial.

We should remark that it is impossible to extract all the editors of each journal automatically, so it is necessary to perform this process manually. This is due to the storage format of the editorial board of each journal, which ranges from highly structured HTML web pages to PDF documents. Therefore, it is infeasible to develop a programming code to extract such information.

The editorial board of the journal *Physikalische Medizin Rehabilitations Medizin Kurort Medizin* was not accessible. Therefore, this journal was excluded from the analysis, leaving the number of journals at 82. Once the database was completed, we proceeded to infer the gender of the editors based on first names.

### 3.2. Inference Process of the Editor's Gender

Once the name of the corresponding editor had been collected, the next step was to infer the gender of that editor taking into account his/her first name. The initial database consisted of almost 5,000 records and a one-to-one search of the gender of the editors on the website is a very time-consuming task.

The process of determining the gender of the editor, knowing his or her first name, was carried out using the *Gender data* package included in the R statistical software (https://www.r-project.org/) version 4.0.5. A similar tool called *genderize.io* in R was previously used by several authors (Refs. [15, 22, 23], among others) to infer people's gender.

Its objective is to classify character strings into gender categories. To classify these character strings, historical datasets (such as censuses) are used. A census collects certain information about the inhabitant, such as name and gender. Many of these censuses are openly available, and R uses them to infer the gender associated with a character string. This package gives a probability to be male or female given a character string. The package *Gender data* (and more specifically the *gender* function) uses the U.S. Census or Social Security listing from 1932 to 2012 to assign this probability. For each first name provided, *Gender data* returns the corresponding probabilities of gender based on frequency counts of it in the census.

The main criticism of these data sources is their liability of the data [24]. The database behind the *genderize.io* draws on data from numerous public profiles, although neither the exact number of sources nor the total number of profiles has been revealed. Also, there is no guarantee that each profile has valid and reliable data in its first name and gender fields. However, it is safe to assume that most people give true information about their gender and given name. Another way to ensure the gender is using those given names that were also entered in other social media profiles, so they seem to be confirmed by other users.

Some first names are unisex. The unisex category means that this first name corresponds to both the male and female gender. For example, “Andrea” is typically a woman's name in the United States, but a man's name in Italy. Some examples of these unisex names found in the database are “Ja,” “Robin,” “Stephane,” and “Toni,” among others. In these cases, the gender inference method does not work properly and the gender is assigned manually performing a direct search of this editor on the website. As a matter of fact, we have decided, by using previous studies, that the gender of an editor is clearly identified when the probability associated with his/her first name exceeds 0.85.

### 3.3. Theoretical Statistical Framework

The method of hypothesis testing was used to analyze the previously explained database obtained by manual search. This method consists of using significance tests to determine the likelihood that a statement is true, and at what likelihood we would accept the statement as true. There is a deep mathematical background behind all this that needs to be well understood. While understanding the mathematical concepts that go into the formulation of these tests is important, the knowledge of how to appropriately use each test is equally important. Once the data are collected, a schema of the test of hypotheses has the following steps:

1. To determine a critical region of size *α* using the sampling distribution of an appropriate test statistic.
2. To determine the value of the test statistic from the sample data.
3. To check whether the value of the test statistic falls within the critical region; if so, the null hypothesis is rejected in favor of the alternative hypothesis, and if not, the null hypothesis is accepted.

## 4. Statistical Analysis and Results

**Figure 1:**
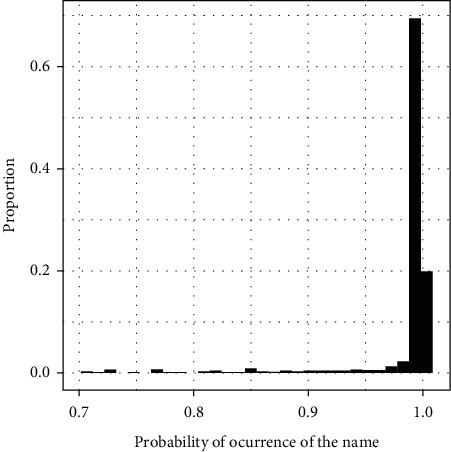
Probabilities of gender based on frequency counts. In this graph, we show the probabilities of occurrence of each name based on the R package *Gender data*. Notice that the vast majority are above 0.9, which means a greater accuracy.

Figure 1 shows the corresponding probabilities of gender based on frequency counts. We accepted the predicted gender for any editor having a probability of gender greater than 0.85. Below this threshold, we manually checked the gender of the editor. In our database, considering 4,596 editors and following this procedure, we found that 23–13% of the 4,596 editorial positions are held by women.

**Figure 2:**
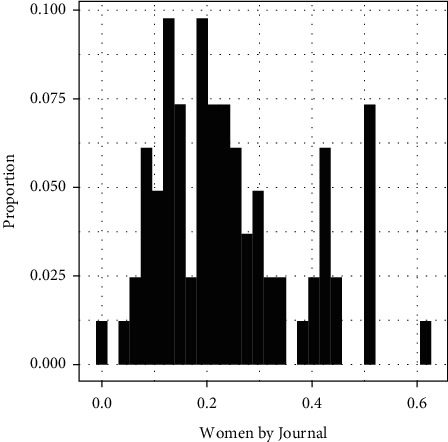
Proportion of women editors by journal. The X-axis represents the different journals. It is shown that the highest proportion (with respect to men) of women editors in a journal is below 0.1, which shows the serious in equality.

Figure 2 shows the distribution of journals by the proportion of editorships held by women.

The *Z* test for the difference between two proportions [25] is used to analyze whether there are significant differences between the percentage of women editors (23.13%) and men editors (76.87%). Obviously, significant differences were found (*p*-value <0.002). This test has been considered because the sample size is greater than 30, so the data are approximately normally distributed. To compare two proportions to see whether they are the same, the hypothesis is as follows:

- The null hypothesis for the test is that the proportions are the same.
- The alternate hypothesis is that the proportions are not the same.

Table 2 shows the average and median number of members that make up each publisher or journal editorial board. The journal with the minimum number of editors is *Exercise Immunology Review*, which is the only journal from the editorial *W.W.F. Verlagsge sell schaft GMBH*.

**Figure 3:**
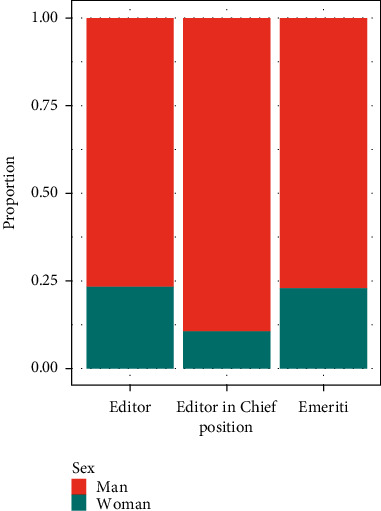
Proportion of women and men editors by position.

In the 82 journals of the 39 editorials in the database, 11 different types of positions (“DeputyEditor,” “Editor,” “Editor in Chief,” “Editorial Assistant,” “Editorial Board,” “Emeriti,” “Executive Editor,” “Managing Editor,” “Publishing Assistant,” “Social Media Editor,” and “Consulting Editor”) were found within the editorial board. For the sake of simplicity, the editors of the dataset have been grouped into 3 categories: Editor, Editor in Chief, and Emeritus.  Figure 3 highlights the discrepancies between the percentages of women in each position.

### 4.1. Proportion of Women Editors on the Editorial Board

The whole proportion of women editors on the editorial board has been calculated taking into account the 82 journals analyzed in the category *Sport Sciences*. In the rest of this article, this proportion is denoted by *p*_0_, and it is equal to 0.2312881.

Next, a proportion test was performed to find significant differences between the proportion of women editors in the editorial board by publisher and the whole proportion of women editors in the editorial board for the 82 journals.

**Figure 4:**
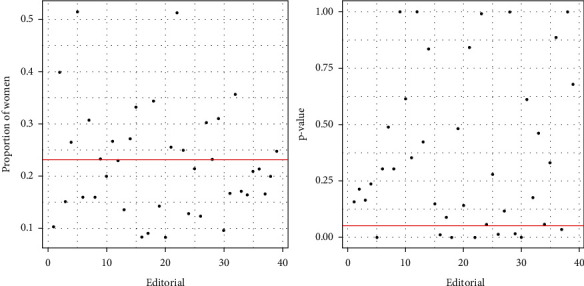
Proportion of women editors and *p*-value according to the publisher.

**Figure 5:**
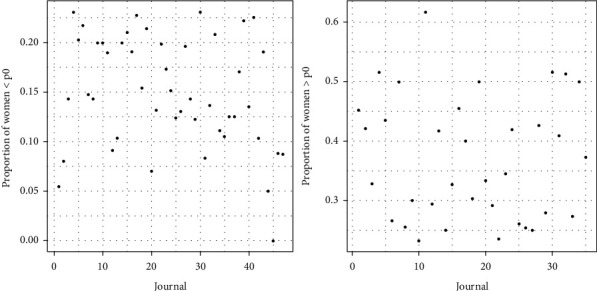
Proportion of women editors on the editorial board.

**Figure 6:**
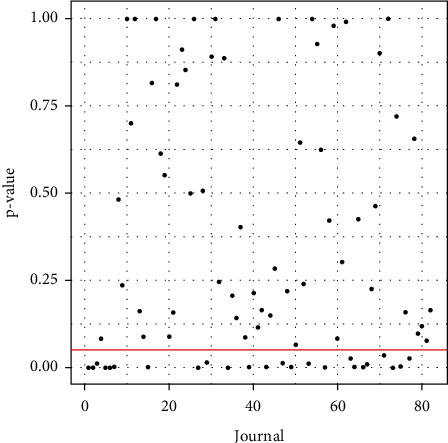
Associated *p*-values.

**Figure 7:**
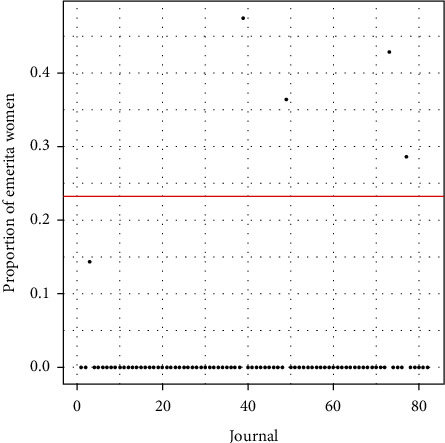
Proportion of Emerita women. On the editorial board (*p*_0_=0.23). A *p*-value of 0.01925 was obtained, so obviously there are significant differences between the total proportion of women editors and the proportion of women EiCs.

Figures 4–7 are shown according to the list of journals from 1 to 82 and according to the list of publishers from 1 to 39 in alphabetical order on the *X*-axis as shown in the Appendix.

### 4.2. Comparison among Publishers

A comparison was performed between the proportion of women editors on the editorial boards (*p*_0_) and the proportion of women editors taking into account the 39 editorials in the database. The test of comparison of proportions is used to find significant differences between the two proportions. Tables 3 and 4 represent the names of the publishers with a proportion of women editors below and above *p*_0_, respectively.  Figure 4 shows the proportion of women editors on the editorial board versus publishers and its corresponding p-values compared to *p*_0_. The red line represents the significance value 0.05 and *p*_0_.

#### 4.2.1. Comparison between Journals

Figure 5 shows the journals whose proportion of women editors in the editorial board exceeds (47 journals) and does not exceed (35 journals) *p*_0_.

Among the 47 journals whose proportion of women editors is below *p*_0_, there are 8 journals with a proportion significantly below *p*_0_ (*p*-value *<*0.05, see Figure 6 for the *p*-values). The journal with the lowest proportion of women editors is *Sportverletz Sport schaden*. This proportion is 0; that is, no woman serves as editor of this journal. In addition, the journals whose proportion of women editors is significantly lower than *p*_0_ are shown in Table 5.

Among the 35 journals whose proportion of women editors is greater than *p*_0_, 16 journals show a significantly greater proportion than *p*_0_ (*p*-value *<*0.05, see Figure 6 for the *p*-values). The journal with the highest proportion of women editors is the *Journal of Aging and Physical Activity*. Other journals with a high proportion of women editors are shown in Table 1.

Finally, Figures 1 and 2 plot the distribution of the proportion of women editors in the 82 journals and the associated probabilities of the first names in the database.

### 4.3. Proportion of Emerita Women Editors

Analyzing the 4,596 editors included in the database, 82 of these editors hold the position of Emeritus that corresponds to 1.78%. Of the total number of Emeritus Editors, 19 are women compared to 63 men, which means that the percentage of Emerita women is 23.17%.

Figure 7 shows the journals with the highest levels of women defined as “Emerita.” The red line in Figure 7 corresponds to the proportion of women Emerita Editors per journal, 0.2317. These journals are

- *Adapted Physical Activity Quarterly.*
- *Journal of Teaching in Physical Education.*
- *Journal of Sport Management.*
- *Sociology of Sport Journal.*
- *The Sport Psychologist.*

As for the relationship between the value *p*_0_ and the proportion of women Emerita editors (0.2317), no significant differences were found (the *p*-value is greater than 0.05).

### 4.4. Proportion of Women EiCs

Another important aspect of this study is to analyze the number of women in top leadership positions on journal editorial boards. In this case, this section focuses on the position of Editor in Chief (EiC).

Comparing the total proportion of women editors and the proportion of women editors holding the position of EiC, the results show that 23% of the editors are women, whereas only 10 out of 97 EiCs are women, which corresponds to 10.3%.

The test of the comparison of proportions was again performed to verify whether there were significant differences between the proportion of women editors in the position of EiC (0.103) and the total proportion of women editors.

The magazines whose Editor in Chief is a woman are the following:

- *Knee.*
- *Journal of Orthopaedic & Sports Physical Therapy.*
- *Psychology of Sport and Exercise.*
- *Journal of Sport Management.*
- *PM & R.*
- *Journal of Sport Rehabilitation.*
- *Motor Control.*
- *Journal of Motor Behavior.*
- *Journal of Applied Physiology.*
- *Applied Physiology, Nutrition and Metabolism.*

To ascertain the influence that a woman EiC might have on the gender composition of the editorial board of a journal, we analyzed whether there were significant differences between the proportion of women editors on the editorial board in each of these journals and the total proportion of women editors, *p*_0_. Statistically significant differences were found in the journals *Journal of Orthopaedic* & *Sports Physical Therapy* (*p*=0.0253), *PM*&*R* (*p*=0.0143), and *Journal of Sport Management* (0.0102), whose estimated proportions of women on the editorial board are 50%, 42.6%, and 41.9%, respectively.

We highlight that of the five women holding the position of Editor in Chief of journals in the first and second quartiles, two share this position with men. In the third and fourth quartiles, no women Editor in Chief has such a shared position.

### 4.5. Women Editor Ratio by Quartiles

**Figure 8:**
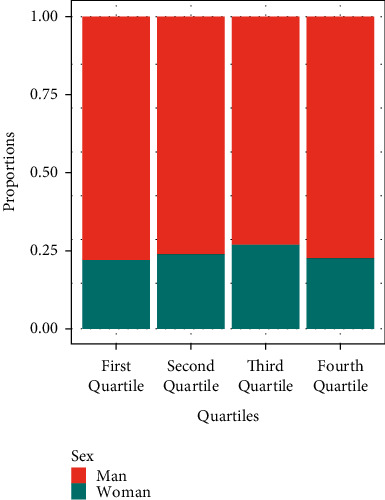
Proportion of women and men editors in each quartile.

This section focuses on analyzing possible significant differences between the total proportion of women editors and the proportion of women editors in the different quartiles (*Q*_1_, *Q*_2_, *Q*_3_, and *Q*_4_). For that, we pooled together all the journals in our dataset belonging to each quartile (this classification of the quartiles is based on the impact factor of 2018).  Figure 8 shows the corresponding proportions of women editors pooled by quartiles.

Table 6 shows the corresponding proportions of women editors and the *p*-values obtained after performing the test of proportion.

A proportions lightly below 0.23 was found in the first and the fourth quartiles (proportions equal to 0.218 and 0.223, respectively). Both in the first and in the fourth quartile, a *p*-value higher than the significance level 0.05 was obtained, which leads us to the conclusion that there are no significant differences between the proportion of women editors in journals of the first and fourth quartile and the total proportion of women editors *p*_0_=0.2312881.

In the case of the second quartile, we obtained an estimated proportion of women editors of 0.237 and a *p*-value*>*0.05, so there would be no statistically significant difference with *p*_0_.

However, in the third quartile, the percentage of women on the editorial board was 26.68%. It was checked by the previous test of comparison proportions if this proportion was significantly higher than *p*_0_. When the test was performed, a *p*-value equal to 0.0082 was obtained, so the proportion of women editors on the editorial board of journals in the third quartile was significantly higher than the total proportion of women editors on the editorial board.

### 4.6. Correlation with Impact Factor

The Pearson coefficient was calculated as the measure of linear dependence between two quantitative random variables to analyze the relationship between the impact factor and the proportion of women editors on the journal editorial boards. The value obtained for this correlation coefficient was 0.0255 (close to 0), so there is no significant correlation between the impact factor of the journal and the proportion of women editors on the editorial board. Another coefficient that measures the strength and direction of association between two ranked variables, not necessarily linear, was calculated. This coefficient is called the Spearman coefficient, and its value for this case is 0.06137443; so, the results and conclusions are similar to those obtained with the Pearson coefficient.

**Figure 9:**
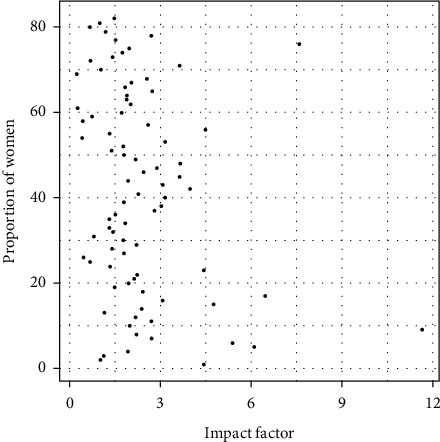
Impact factor of the journal and proportion of women editors.

Figure 9 shows that a clear relationship was not found between the impact factor variables and the proportion of women editors on the editorial board of the 82 journals.

In Sport Sciences journals, dependencies between the authority of editors and journal impact factors were found, for example, in Kay et al. [26]. Besides, there is a positive correlation between the rank of journals and the presence of women on the editorial board in some other disciplines (Metz and Harzing [21]).

## 5. Conclusions

This study analyzes the gender composition of the editorial boards of 82 journals included in the *Sport Sciences* category. Of these 82 journals, only one had no women on its editorial board, which corresponds to a percentage of 1.2% of journals with no women on the editorial board. This proportion is lower than the proportion shown in Ref. [15], where 11.72% of mathematics journals included in the JCR had no women on their editorial board.

In the category of Sport Sciences analyzed in this article, the total proportion of women editors was *p*_0_=0.23, which is higher than the total percentage of women editors on the editorial boards of mathematics journals (8.9%) [15] and medicine journals (15.9%) [16, 17]. However, it is lower than the total percentage of women on the editorial board of journals in psychiatry (30.4%) [18] and political sciences (26%) [19].

This study found no significant differences between the proportion of women editors and the proportion of Emerita women on the editorial board. The position of Emeritus may be a snapshot of the past on a journal editorial board. Therefore, the fact that the proportion of women editors remains at 23% indicates that the current situation is similar to the past (in terms of gender representation on editorial boards).

No correlation was found between the impact factor and the proportion of women editors. This is in line with the fact that significant differences were found only in the third quartile between the proportion of women editors on the editorial board of journals of these quartiles and the total proportion of women editors on the editorial board *p*_0_=0.23.

Moreover, nine journals have a woman as EiC, corresponding to a total of 10.31% women Editors in Chief. This percentage is much lower than that indicated in the study carried out by Ref. [1] in Spanish scientific journals. Of the nine journals with a woman Editor in Chief, in 66% of them, the proportion of women editors on the editorial board is significantly higher than the total proportion of women editors on editorial boards *p*_0_. In the remaining 33%, the proportion of women editors does not present significant differences with respect to the total proportion of women editors on editorial boards. That is, in all those journals whose proportion of women editors on the editorial board is significantly lower than *p*_0_, the Editor in Chief is a man.

## 6. Discussion

Women remain under-represented in Sport Sciences journals. The results of our study were consistent across 4,596 editors of 82 different journals in the category *Sport Sciences*. Adjustment for Editor in Chief, Emerita Editor, Editor in General, and the comparison between publishers and journals did not eliminate gender differences. Nevertheless, differences persisted across every academic field in different ways: 8.9% of women on the editorial board of mathematics [15], 15.9% in medicine [16, 17], but 26% and 30.4% in political sciences [19], and psychiatry [18].

Our analyses comparing editorial board positions suggest that women have lost ground in terms of promotion. This finding confirms those from other recent studies. The study results that were published in 2018 showed that over 17 years, among 1,273 faculty members at 24 U.S. medical schools, women were less likely than men to attain leadership positions such as dean, associate dean, provost, and department chair, even after adjustment for publication-related productivity.

In this analysis representing data from years 2020 to 2021, women were found to be less likely than men to be members of the editorial board in a journal, especially involving leadership positions. In general, it has been found that a magazine with a woman Editor in Chief is more likely to have more women on its editorial staff.

Our findings indicate that editorial positions appear to be failing in eliminating gender differences in promotion. Women are under-represented both among Emerita and Editor in Chief positions, which are role models for career advancement and on editorial boards of Sport Science journals, which prioritize areas of research and determine which authors will have their work published. Although this is a descriptive study, it identifies a very relevant social fact at present “the under-representation of women in science.” Moreover, the novelty lies in the fact that it is studied in an area that had never been studied before: the editorial boards of scientific journals in the category of Sport Sciences. Future studies should examine the effect of the intersection of race, ethnic group, and gender on editorial boards of Sport Science journals.

## 7. Limitations and Future Research Lines

As a first limitation, there is a percentage of journal editors, from whom it was impossible to extract their names reliably and truthfully, and therefore, they could not be included in the database. Regarding the first name of the researcher, 1,223 different character chains have been identified. There are character chains that correspond to both male and female names, for example, *“Jan,” “Robin,” “Stephane,” “Toni,” “Tracy,”* and *“Yuri.”* For editors whose first name corresponds to this string of characters, a manual search procedure was performed to find out their gender.

The initial database consisted of 5,177 editors, from which the gender could not be inferred in 581.

Therefore, eliminating duplication, finally the database was reduced to 4,596 editors.

If we focus on the *Gender* tool used in the Methodology section, it should be taken into account that the census used by the R program is of U.S. origin and therefore identifies Anglo-Saxon root names quite accurately, but its reliability is lower if the names are of Latin or Asian origin. There are 795 names with probability 1, and this means that only one person with these names has been found in the U.S. census. An improvement to the inference process used in this study would be to take into account the frequency of the names in the census to assign a given gender probability.

The relationship with the impact factor is very interesting, so we propose it as a future line of research, as we do not have enough information to study it in this article. In the same way, the correlation with the gender of the authors of published papers is very enriching for science but has certain limitations. It should be noted that there is insufficient time to carry out the data collection for each article published in each journal, taking into account the gender of the authors. However, it would be another interesting line for the future if it were done through an automatic platform that optimizes time, instead of using field work, as in our study.

This paper has been limited to the analysis of a single JCR category. It would be of great interest to analyze the gender composition of the editorial board and the comparison between the different categories related to Sport Sciences such as *Hospitality, Leisure, Sport* & *Tourism*, *Education* & *Educational Research*, *Public, Environmental* & *Occupational Health, Environmental Sciences* and Education/*Psychology,* and *Educational Research,* among others.

Finally, it is important to point out that the methodological tool is based on a binary classification of the gender; hence, certain groups such as transsexuals are excluded. In this paper, we limit ourselves to describing the current situation. In future research, it would be useful and interesting to compare the representation of women in editorial positions with their representation in the discipline. There are currently no studies in this area with which to make such a comparison, hence the novelty of our study.

## Figures and Tables

**Table 1:** Journals with a high proportion of women editors.

Journal	Proportion	*p*-value
*Journal of Aging and Physical Activity*	0.6166	<0.001
*ACSM Health and Fitness Journal*	0.452	<0.001
*Applied Physiology, Nutrition, and Metabolism*	0.5156	<0.001
*Sociology of Sport Journal*	0.5128	<0.001

**Table 2:** Size of the editorial board.

	Publishers	Journals
Median	56	44.5
Mean	117.67	56.5
Minimum	10	10
Maximum	775	237

**Table 3:** Publishers with a proportion of women editors with statistical significance *p*_0_.

Publisher	Proportion	*p*-value
*Sage Publications*	0.0964	<0.002
*Georg Thieme Verlag KG.*	0.083	0.00103

**Table 4:** Publishers with a proportion of women editors with statistical significance over *p*_0_.

Publisher	Proportion	*p*-value
*Human Kinetics*	0.3452	*<*0.001
*Canadian Science Publishing*	0.5156	*<*0.001
*JOSPT*	0.5143	<0.001
*Mosby-Elsevier*	0.124	0.0126
*Routledge Journals Taylor* & *Francis LTD*	0.311	0.0142
*W.B. Saunders CO. Elsevier INC.*	0.166	0.0352

**Table 5:** Journals with a women editor proportion lower than *p*_0_.

Journal	Proportion	*p*-value
*Sport Medicine and Arthroscopy Review*	0.054	<0.001
*Journal of Orthopaedic Trauma*	0.0703	<0.001
*American Journal of Sports Medicine*	0.08029	<0.001
*Orthopaedic Journal of Sports Medicine*	0.084	<0.001

**Table 6:** Women editors by quartiles.

	Proportion of women editors	*p*-value
First quartile	0.2184057	0.121
Second quartile	0.237594	0.3272
Third quartile	0.2668513	0.003076
Fourth quartile	0.2234848	0.6839

## Data Availability

The datasets used during the current study are available from the corresponding authors on reasonable request.

## Appendix

### A. Journals in the category “Sport Sciences” in JCR

1. Acsms Health & Fitness Journal.
2. Adapted Physical Activity Quarterly.
3. American Journal of Physical Medicine & Rehabilitation.
4. American Journal of Sport Medicine.
5. Applied Physiology Nutrition and Metabolism.
6. Archives of Budo.
7. Archives of Physical Medicine and Rehabilitation.
8. Arthroscopy-The journal of arthroscopic and related surgery.
9. Biology of Sport.
10. British Journal of Sport Medicine.
11. Clinical Bio-mechanics.
12. Clinical Journal of Sports Medicine.
13. Clinics in Sports Medicine.
14. Current Sports Medicine Reports.
15. European Journal of Applied Physiology.
16. European Journal of Sport Science.
17. Exercise and Sport Sciences Reviews.
18. Exercise Immunology Review.
19. Gait & Posture.
20. High Altitude Medicine & Biology.
21. Human Movement Science.
22. International Journal of Performance Analysis in Sport.
23. International Journal of Sport Nutrition and Exercise Metabolism.
24. International Journal of Sport Psychology.
25. International Journal of Sports Medicine.
26. International Journal of Sports Physiology and Performance.
27. Isokinetics and Exercise Science.
28. Journal of Aging and Physical Activity.
29. Journal of Applied Biomechanics.
30. Journal of Applied Physiology.
31. Journal of Applied Sport Psychology.
32. Journal of Athletic Training.
33. Journal of Electromyography and Kinesiology.
34. Journal of Exercise Science & Fitness.
35. Journal of Human Kinetics.
36. Journal of Motor Behavior.
37. Journal of Orthopaedic & Sport Physical Therapy.
38. Journal of Orthopaedic Trauma.
39. Journal of Rehabilitation Medicine.
40. Journal of Science and Medicine in Sport.
41. Journal of Shoulder and Elbow Surgery.
42. Journal of Sports & Exercise Psychology.
43. Journal of Sport and Health Science.
44. Journal of Sport Management.
45. Journal of Sports Medicine and Physical Fitness.
46. Journal of Sport Rehabilitation.
47. Journal of Sport Sciences.
48. Journal of Sports Science and Medicine.
49. Journal of Strength and Conditioning Research.
50. Journal of Teaching in Physical Education.
51. Journal of the International Society of Sports Nutrition.
52. Kinesiology.
53. Knee.
54. Knee Surgery Sports Traumatology Arthroscopy.
55. Medicine and Science in Sports and Exercise.
56. Medicin a Dello Sport.
57. Motor Control.
58. Operative Techniques in Sports Medicine.
59. Orthopaedic Journal of Sports Medicine.
60. Pediatric Exercise Science.
61. Physical Therapy in Sport.
62. Physician and Sports Medicine.
63. PM & R.
64. Proceedings of the Institution of Mechanical Engineers Part P: Journal of Sports Engineering and Technology.
65. Psychology of Sport and Exercise.
66. Quest.
67. Revista Brasileirade Medicin a do Esporte.
68. Revista Internacional de Medicinay Ciencias dela Actividad Físicaydel Deporte.
69. Research in Sports Medicine.
70. Research Quarterly for Exercise and Sport.
71. Scandinavian Journal of Medicine & Science in Sports.
72. Science & Sport.
73. Sociology of Sport Journal.
74. Sport Education and Society.
75. Sports Health-A Multidisciplinary Approach.
76. Sport Medicine.
77. Sport Medicine and Arthroscopy Review.
78. Sport Psychologist.
79. Sports Biomechanics.
80. Sportver letzung-sport schaden.
81. Strength and conditioning journal.
82. Wilderness & Environmental Science.

### B. Publishers in the category “Sport Sciences” in JCR

1. AdisINTLTD.
2. AmericanPhysiologicalSociety.
3. BMC.
4. BMJ Publishing Group.
5. Canadian Science Publishing.
6. DeGruyter Poland SP Zoo.
7. Edizioni Luigi Pozzi.
8. Edizioni Minerva Medica.
9. Elsevier France.
10. Elsevier Ireland LTD.
11. Elsevier SCILTD.
12. Elsevier Science BV.
13. Elsevier Science INC.
14. Elsevier Singapore PTELTD.
15. Foundation Rehabilitation Information.
16. Georg Thieme Verlag KG.
17. Human Movement SCI.
18. Human Kinetics Publ INC.
19. INST Sport.
20. INT Scientific Information, INC.
21. IOS Press.
22. J.O.S.P.T.
23. Journal Production DEPT.
24. Journal Sports Science & Medicine.
25. Lippincott Williams & Wilkins.
26. Mosby-Elsevier.
27. NATL Athletic Trainers Association INC.
28. RedIris.
29. Routledge Journals, Taylor & Francis LTD.
30. Sage Publications INC.
31. Sage Publications LTD.
32. Shanghai University Sport.
33. Soc Brasileira Medicine Esporte.
34. Springer.
35. Taylor & Francis LTD.
36. University Zagreb, Fac Kinesiology.
37. W.B. Saunders CO.Elsevier INC.
38. W.W. Verlagsge sell schaft GMBH.
39. Wiley.
